# Ghrelin, MicroRNAs, and Critical Limb Ischemia: Hungering for a Novel Treatment Option

**DOI:** 10.3389/fendo.2017.00350

**Published:** 2017-12-13

**Authors:** Joshua P. H. Neale, James T. Pearson, Rajesh Katare, Daryl O. Schwenke

**Affiliations:** ^1^Department of Physiology-HeartOtago, University of Otago, Dunedin, New Zealand; ^2^Department of Cardiac Physiology, National Cerebral and Cardiovascular Center Research Institute, Suita, Japan; ^3^Biomedicine Discovery Institute and Department of Physiology, Monash University, Clayton, VIC, Australia

**Keywords:** critical limb ischemia, peripheral artery disease, ghrelin, microRNAs, angiogenesis, regeneration, vascular disease, no-option critical limb ischemia patients

## Abstract

Critical limb ischemia (CLI) is the most severe manifestation of peripheral artery disease. It is characterized by chronic pain at rest, skin ulcerations, and gangrene tissue loss. CLI is a highly morbid condition, resulting in a severely diminished quality of life and a significant risk of mortality. The primary goal of therapy for CLI is to restore blood flow to the affected limb, which is only possible by surgery, but is inadvisable in up to 50% of patients. This subset of patients who are not candidates for revascularisation are referred to as “no-option” patients and are the focus of investigation for novel therapeutic strategies. Angiogenesis, arteriogenesis and vasculogenesis are the processes whereby new blood vessel networks form from the pre-existing vasculature and primordial cells, respectively. In therapeutic angiogenesis, exogenous stimulants are administered to promote angiogenesis and augment limb perfusion, offering a potential treatment option for “no option” patients. However, to date, very few clinical trials of therapeutic angiogenesis in patients with CLI have reported clinically significant results, and it remains a major challenge. Ghrelin, a 28-amino acid peptide, is emerging as a potential novel therapeutic for CLI. In pre-clinical models, exogenous ghrelin has been shown to induce therapeutic angiogenesis, promote muscle regeneration, and reduce oxidative stress *via* the modulation of microRNAs (miRs). miRs are endogenous, small, non-coding ribonucleic acids of ~20–22 nucleotides which regulate gene expression at the post-transcriptional level by either translational inhibition or by messenger ribonucleic acid cleavage. This review focuses on the mounting evidence for the use of ghrelin as a novel therapeutic for CLI, and highlights the miRs which orchestrate these physiological events.

## Introduction

Peripheral artery disease (PAD) is characterized by the narrowing or occlusion of systemic arteries impeding blood supply to the extremities (1). It is rapidly emerging as a significant global socio-economic burden and currently affects over 202 million people (2). PAD is associated with significant cardiovascular morbidity and mortality (3), plus a severely diminished quality of life (4). PAD is most commonly a result of atherosclerosis and, as such, risk factors for the development of the disease include smoking, diabetes mellitus (DM), hypertension (5), and advanced age (6). With the number of people being diagnosed with DM expected to surpass 640 million by 2040 (7) and the acceleration of global population aging (8), the PAD burden is expected to rapidly increase (2).

At the severest end of the PAD spectrum is critical limb ischemia (CLI), which describes patients with chronic pain at rest, skin ulcerations, and gangrene tissue loss (3). CLI accounts for 11% of patients with PAD, increasing to 20% in patients over 70 years of age (9). The prognosis of CLI is very poor; at 6 months post-diagnosis, patients have a 25–40% chance of lower limb amputation and ~20% chance of mortality (10). The quality of life for CLI patients is also severely diminished, similar to those with terminal cancer (11). Management of CLI includes risk factor modification and aggressive pharmacological therapy, accompanied with either an endovascular or surgical intervention (12, 13). However, revascularisation as a primary treatment approach is expensive, labour-intensive, and is often met with poor success (14). Unfortunately, up to 50% of CLI patients are not candidates for surgery due to extreme tissue damage, diffuse atherosclerotic disease, and co-morbidities (15, 16). This subgroup of patients is referred as “no-option” patients and is the focus of research for novel therapies.

Therapeutic angiogenesis is emerging as a potential treatment approach for “no-option” CLI patients by stimulating neovascularization, improving limb perfusion, and aiding tissue regeneration (17). Angiogenic agents, such as gene or cell therapy, have been the focus of investigation, with the aim of inducing a pro-angiogenic milieu in the affected ischemic limb. Regrettably, the results from clinical trials using such agents have shown little clinical benefit regarding primary outcome measures (i.e., patency, amputation-free survival, major limb adverse effects) (18–20). This highlights both the complexity of therapeutic angiogenesis and the need to develop new agents for the management of CLI.

The 28-amino acid peptide hormone ghrelin, first discovered in 1999 as the endogenous ligand for the growth hormone secretagogue receptor (GHS-R) (21), has recently been proposed as a novel therapeutic for CLI (22–24). The GHS-R is a G-protein coupled receptor, ubiquitous throughout the cardiovascular and autonomic nervous systems (25, 26), which plausibly accounts for the diverse range of effects ghrelin has on cardiovascular function (25, 26).

This review presents the concepts of therapeutic angiogenesis and highlights the limitations associated with advancing current treatments for CLI. Furthermore, the emergence of ghrelin as a novel therapeutic for CLI is explored and the molecular mechanisms that underpin ghrelin’s beneficial actions highlighted.

## Pathophysiology of CLI

The pathological events which lead to the presentation of CLI are multifactorial, complex, and beyond the scope of this review [reviewed in Ref. (24)]. The underlying pathological events leading to CLI are macro- and microvascular circulation defects, resulting in diminished arterial perfusion. Consequently, the metabolic requirements of the distal tissue outweigh the oxygen and nutrient supply. Although the aetiology of CLI can be vasculitis, thromboembolic disease, trauma, popliteal entrapment, cystic adventitial disease, thromboangiitis, and Buerges disease, it is most commonly associated with diffuse atherosclerosis (27, 28). The compensatory mechanisms against initial ischemia involve angiogenesis and arteriogenesis to increase blood flow to the affected tissue. However, these compensatory mechanisms are ineffective in CLI. Distal arterioles respond to ischemia by decreasing wall thickness, maximally vasodilating, subsequently becoming unresponsive to provasodilatory stimuli; a term referred to as vasomotory paralysis (29). Moreover, arterioles exhibit an inability to control vascular resistance as a result of blunted myogenic autoregulation (29). Combined, these factors lead to an orthostatic-dependent increase in hydrostatic pressure in the distal part of the limb, resulting in the development of oedema (30). Furthermore, chronic inflammation and the production of free radicals further exacerbate endothelial dysfunction. Endothelial damage, inappropriate platelet activation, and leukocyte adhesion contribute to a microthrombi formation, distal oedema, and tissue damage.

## Therapeutic Angiogenesis

Angiogenesis is a precisely orchestrated process of events which is essential for optimizing or restoring organ perfusion. Artificially cultivating this complex process of events is the ultimate goal of therapeutic angiogenesis (31). Therapeutic angiogenesis is essentially the promotion of new blood vessel growth in an ischemic tissue bed to supply a local demand for oxygen and aid tissue recovery. This new vasculature can be induced by three distinct means: angiogenesis, arteriogenesis, and vasculogenesis. Angiogenesis is defined as the formation of new capillaries from the pre-existing vasculature (32). It can occur by two distinct mechanisms: intussusception and sprouting (33–35). Intussusception defines the process in which transluminal tissue pillars develop in the capillaries resulting in the vessel splitting, thus creating new vasculature entries and increasing the vascular network (35). Sprouting angiogenesis is the growth of new capillary vessels from a pre-existing vessel and occurs in the following stages: proteolytic degradation of extracellular matrix, endothelial cell migration and proliferation, tubulogenesis, vessel fusion, vessel pruning, and functional maturation (32). Arteriogenesis is characterized by the growth of collateral arteries, in addition to the remodeling and enlargement of the pre-existing arterioles (36, 37). Finally, vasculogenesis is defined as the *de novo* formation of a primitive vasculature from endothelial precursor cells, which is traditionally considered only to take place during embryogenesis (38). These three events, which to some extent occur naturally in response to ischemia, are influenced by genetic factors but are also the targets of therapeutic agents.

## Therapeutic Potential of Angiogenic Factors

In the Western nations, it is estimated that over 300 million people may benefit from a pro-angiogenic therapy (39). Despite considerable research to develop a pro-angiogenic agent for the effective treatment of CLI, no therapeutic agents have been approved by the Medicines and Healthcare Products Regulatory Agency of the United Kingdom or the Food and Drug Administration of the United States of America. However, in Russia, a plasmid DNA gene product encoding vascular endothelial growth factor (VEGF) 165 “Neovasculgen” (40) is approved for clinical use in the treatment of PAD. While this agent is approved in Russia, a recent meta-analysis of randomized control trials shows no consistent benefit of any gene therapy for promoting therapeutic angiogenesis in PAD (20).

Extensive research has identified numerous pro-angiogenic agents for CLI (Table 1 contains a partial list of the studies). However, to date, there is a clear disparity between pre-clinical trials and large randomized control trials. Few clinical trials report long-term positive effects or clinically significant results (18, 41). These disappointing findings with current angiogenic agents can be attributed to numerous factors, including suboptimal delivery strategies (42), disease mediated dysfunction (43), methodological differences, and pre-clinical models which lack common CLI risk factors (41). To ensure the future success of therapeutic angiogenesis, it is imperative that clinical methods are standardized (i.e., angiogenic agent selection, delivery method, *in vitro* tracking) and focus on more applicable pre-clinical models which better mimic human CLI. Despite potentially promising outcomes in the early stages of some pre-clinical studies, current angiogenic agents had limited long-term success. Due to this limited success, hormone therapy is emerging as a novel treatment strategy.

**Table 1:** Factors with an angiogenic potential.

Reference	Year	Cohort size	Patient characterization	Agent	Route of administration	Follow up
Teraa et al. (44)	2015	160	Critical limb ischemia (CLI)	BMMNC	IA	9 months
Gupta et al. (45)	2013	20	CLI	BMMSC	IM	24 weeks
Szabo et al. (46)	2013	20		Ves-Cell	IM	22.6 months
Lara-Hernandez et al. (47)	2010	28	CLI	EPC	IM	14.7 months
Kinoshita et al. (48)	2012	17	CLI	G-CSF-mobilized CD34+	IM	52 months
Kusumanto et al. (49)	2006	27	Diabetic CLI	VEGF	IM	14 weeks
Belch et al. (50)	2011	259	CLI	FGF	IM	12 months
Shigematsu et al. (51)	2010	40	CLI	HGF	IM	12 weeks
Rajagopalan et al. (52)	2007	34	CLI	HIF-1α	IM	12 months

*BMMNC, bone marrow mononuclear cells; BMMSC, bone marrow mesenchymal stem cells; EPC, endothelial progenitor cell; G-CSF, granulocyte-colony stimulating factor; VEGF, vascular endothelial growth factor; FGF, fibroblast growth factor; HGF, hepatocyte growth factor; HIF-1α, hypoxia-inducible factor-1α*.

### Ghrelin

**Figure 1:**
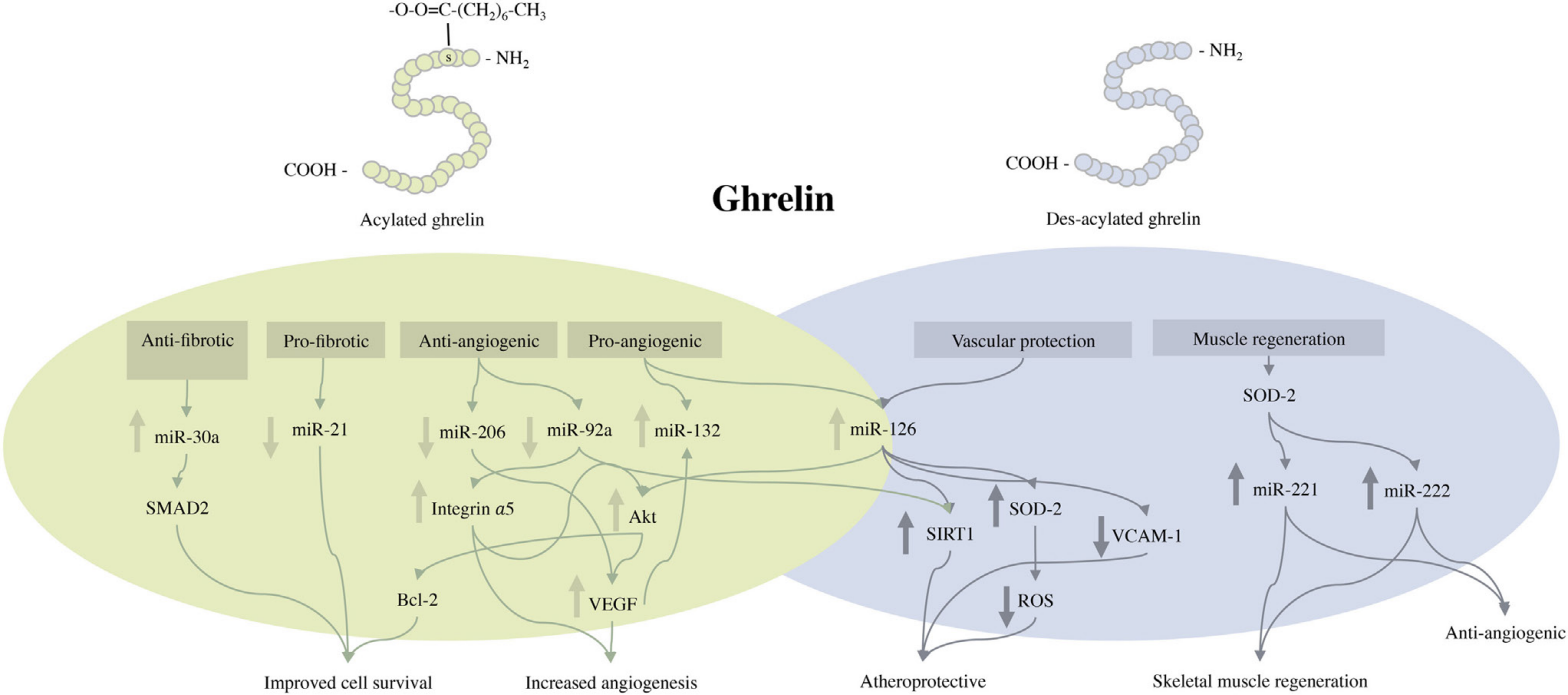
Schematic representation of the structure of acylated and des-acylated ghrelin and the proposed molecular events activated by exogenous ghrelin treatment following critical limb ischemia. miR, indicates microRNA; VEGF, vascular endothelial growth factor; Akt, protein kinase B; Bcl-2, B-cell lymphoma 2; SIRT1, surtuin 1; VCAM-1, vascular cell adhesion molecule 1; SOD-2, superoxide dismutase-2; ROS, reactive oxygen species.

The hormone ghrelin was first isolated from rat stomach in 1999 and was subsequently identified as the endogenous ligand for the GHS-R type 1a (21). Ghrelin is predominantly produced by P/D1-like (X/A-like cells in the mouse) cells in the oxyntic glands of the gastric mucosa, but is also expressed in a smaller amount in other tissues including the myocardium, arteries, and veins (25, 26). Ghrelin circulates in two distinct forms: acylated ghrelin (AG) and des-acylated ghrelin (Des-AG). AG possesses a novel octanoylation of serine-3, promoted by ghrelin *O*-acyltransferase (Figure 1). This is considered essential for facilitating the binding of the peptide to the GHS-R and eliciting its physiological functions (53). Des-AG lacks GH-secretagogue activity and does not bind to the GHS-R type 1a. The mechanisms by which Des-AG exerts its biological functions remain largely unknown, as its target receptor is yet to be identified. Emerging evidence from the literature consistently advocates the therapeutic potential of ghrelin as a novel strategy for the treatment of various metabolic and cardiovascular disorders. These include anorexia, sarcopenia, cardiomyopathy, CLI, inflammatory disorders, metabolic syndrome, epilepsy, and neurodegenerative disorders (54–60).

## Ghrelin and CLI

### Angiogenic Potential

Ghrelin has been shown to have a pro-angiogenic potential in several studies (22, 61–64), but limited potential in others (65–67). Ghrelin’s angiogenic potential was first shown in human microvascular endothelial cells (HMVEC) at a concentration of 0.1 nM. At this concentration, it promoted migration, proliferation, and angiogenesis through phosphorylation and activation of the mitogen activated protein kinases ERK2, which regulates endothelial cell function (63). The same research group has also demonstrated that AG at 1 nM significantly reverses age-related impaired angiogenesis in HMVECs. This is mainly through the activation of the MAPK/ERK2 mitogenic signalling pathway, a central pathway for angiogenesis which promotes endothelial cell motility and survival (62). Using cardiac microvascular endothelial cells, AG at concentrations of 10 nM significantly stimulated proliferation, migration, and angiogenesis *in vitro*. This was through the GHS-R1a type 1a-mediated kinase MEK and extracellular signal-regulated kinase ERK, in addition to the most commonly studied pro-survival phosphatidylinositol 3-kinase/protein kinase B (PI3K/Akt) pathway, which regulates numerous cellular processes, including cell cycle, angiogenesis, and apoptosis (68). Taken together, these findings indicate exogenous ghrelin administration can activate several independent angiogenesis signalling pathways (61).

In a rat model of chronic myocardial ischemia, following permanent occlusion of the left anterior descending artery, AG (100 µg/kg, twice daily for 4 weeks) increased VEGF expression, inhibited apoptosis, and increased angiogenesis in the myocardial infarct and peri-infarct zones (64). In a murine model of CLI, AG (150 µg/kg, daily for 2 weeks) promoted angiogenesis through the up-regulation of pro-angiogenic microRNAs (miRs)-126 and-132, while preventing the activation of antiangiogenic miRs-92a and -206 (22).

Interestingly, Des-AG (100 µg/kg daily for 2 weeks) also exerted a protective vascular effect in a murine model of CLI, evident from a comparatively similar blood vessel density in the ischemic versus contralateral (non-ischemic) limbs (69). However, Des-AG did not change large vessel perfusion or induce neovascularization, possibly due to its up-regulation of antiangiogenic miRs-221 and -222 (70). This suggests Des-AG may limit adverse changes in the vasculature in response to ischemia, rather than initiate angiogenesis (69, 71).

Although the majority of studies advocate the angiogenic potential of AG, conflicting reports from one research team propose that AG may have antiangiogenic properties. This is based on the observation that AG (10 nM) impeded the *in vitro* activation of angiogenesis induced by fibroblast growth factor-2 in human umbilical vein endothelial cells (HUVEC), and in rat neuromicrovascular endothelial cells, *via* inhibition of tyrosine kinase and MAPK pathways (65, 67).

The discrepancy between studies, especially *in vitro* studies, concerning the angiogenic properties of ghrelin is unquestionably confounded by experimental factors. These include differing cell types, culture conditions, different concentrations, and differing methodological approaches. However, it is clear from *in vivo* animal models of MI and CLI that ghrelin exerts beneficial effects through vascular protection and neovascularization.

### Skeletal Muscle and Peripheral Nerve Regeneration

Impaired perfusion of the lower limbs results in significant atrophy of the calf muscle and increased fat composition, causing severe functional impairment (72). Circulating levels of ghrelin are reduced in diseases of muscle wasting (650 vs. 899 pg/mL) (73). Yet, the therapeutic administration of exogenous ghrelin in a murine model of muscular atrophy induces Akt^S473^ phosphorylation- a direct anti-atrophic signalling pathway in skeletal muscle, blocking skeletal muscle atrophy (74). In murine models of CLI, Des-AG has been shown to induce skeletal muscle regeneration through increased superoxide-dismutase-2 (SOD-2) induced expressions of miRs-221 and-222 (69). AG has been demonstrated to significantly up-regulate anti-fibrotic miR-30a, inhibit pro-fibrotic miR-21, and inhibit miR-206 resulting in increased proliferation of myocytes and aiding in tissue repair (22).

In patients who have DM as a comorbidity with CLI, peripheral neuropathy commonly leads to a delayed clinical presentation of the disease, resulting in severe muscular and vascular damage (75). Several studies have reported that AG administration (300 nmol/kg) ameliorated polyneuropathy in rodent models of diabetic polyneuropathy, evident by increased nerve conduction velocities and temperature sensation (76, 77). Although the mechanisms by which ghrelin alleviates polyneuropathy remain to be identified, these findings further support ghrelin as a potential treatment option for an otherwise intractable disorder. Recently, these findings have also been confirmed in a human study (78), with ghrelin treated patients (1.0 µg/kg for 14 days) having increased motor nerve conduction velocity of the posterior tibial nerve compared with controls. Total symptom score also significantly improved in the treated group compared to controls, suggesting that ghrelin may be a novel therapeutic option for diabetic polyneuropathy. However, a double-blind, placebo-controlled trial is needed in the future.

The results from these studies demonstrate that ghrelin acts to protect against skeletal muscle atrophy, neuropathy, and importantly, aids in muscle regeneration.

### Anti-inflammatory and Antioxidant Actions

The diminished supply of blood to the periphery in CLI and the highly oxidative environment result in severe skeletal muscle damage. SOD-2 is the main antioxidant defence against reactive oxygen species (ROS) production and has been shown to be inhibited in skeletal muscle affected by CLI (79). The two isoforms of ghrelin, AG and Des-AG, have been shown to exert potent anti-inflammatory and antioxidant effects throughout the vasculature.

AG has been shown to exhibit anti-apoptotic effects through the strong activation of Akt and ERK, mediated by the PI3K and MAPK pathways (80), and to inhibit pro-inflammatory cytokine production *in vitro* through the inhibition of nuclear factor-kappa B (81). AG has been shown to inhibit apoptosis, and blunt ROS production in HUVECs treated with high glucose, replicating diabetic vascular complications, *via* the PI3K/Akt pathway (82). AG (10^6^ M) inhibits advanced glycation end products-mediated cell apoptosis *via* the ERK1/2 and PI3K/Akt pathways (83). Moreover, ghrelin has been shown to inhibit vascular oxidative stress through inhibition of vascular NAD(P)H oxidases (84).

Des-acylated ghrelin has been shown to protect against oxidative stress-induced apoptosis through the class III histone deacetylase sirtuin (SIRT1), a nicotinamide adenine dinucleotide dependent histone/protein deacetylase signalling pathway (85). Activation of this pathway has been shown to reduce apoptosis and protects endothelial progenitor cells from ROS-mediated damage under conditions of diabetes (86, 87). In a murine model of CLI and glucose intolerance, Des-AG was also demonstrated to rescue miR-126 expression, a regulator SIRT1 and SOD-2, leading to improved oxidative stress levels and improved recovery (71).

### Dysregulation of Ghrelin in Atherosclerosis

Atherosclerosis underlies most of CLI. Circulating ghrelin concentrations have been shown to be reduced in several atherosclerotic diseases (88–92). To date, the circulating ghrelin levels in patients with PAD or CLI remain unknown. However, in diseases with a similar aetiology, ghrelin levels have been shown to be dysregulated. These include patients with unstable angina (1.04 ± 0.08 ng/mL), acute myocardial infarction (1.07 ± 0.11 ng/mL), and asymptomatic coronary artery disease (2.1 ± 0.8 ng/mL), which have all been shown to have lower total ghrelin concentrations compared to controls (6.2 ± 4.1 ng/mL) (88). In addition to total ghrelin, low circulating levels of Des-AG (~78.2 fmol/mL) have been shown to be associated with increased risk of cardiovascular events in older hypertensive individuals (89). However, further studies are warranted to elucidate the pathogenic mechanisms underlying this association, giving clarity to the clinical value of the measure. In contrast, high plasma concentration of ghrelin has been shown to protect against coronary heart disease (90), and AG is significantly higher in patients with ischemic heart disease (coronary artery disease) compared to controls (valvular heart disease; 32 ± 3 vs. 16 ± 2 pg/mL) (91), potentially demonstrating a role for ghrelin in auto- and paracrine self-protective mechanisms of the ischemic heart. In addition, total circulating ghrelin concentrations are decreased in patients with acute ischemic stroke compared to age- and sex-matched controls (3.47 ± 1.44 vs. 5.93 ± 2.78 ng/mL) (92).

In a murine model of atherosclerosis, AG (10^−9^ mol/kg/day for 4 weeks) has also been shown to reduce the formation of atherosclerotic lesions, increase plaque stability, ameliorate activation of endoplasmic reticulum stress, and decrease intima-media thickness (93, 94).

Collectively, this evidence from the literature suggests that plasma ghrelin concentration may be a novel prognostic marker of the extent and severity of various forms of atherosclerotic disease. Additionally, ghrelin may be a promising adjunct therapy for the treatment of CLI and the underlying mechanisms of CLI, atherosclerosis (95).

### Vascular Calcification Reduction

Vascular calcification is characterized by the progressive enlargement of calcium deposits in the major arteries, and is an independent risk factor for CLI (96). Serum ghrelin level decreases with the severity of the tibial artery stenosis in diabetic patients (mild stenosis, 167.71 ± 16.73; moderate stenosis, 105.72 ± 10.51; severe stenosis/occlusion, 53.11 ± 5.65 ng/mL) (97). *In vivo* and *in vitro* models of vascular calcification consistently demonstrate that ghrelin peptide levels and mRNA expression are significantly reduced during calcification. However, treatment with exogenous AG (10^−8^–10^−5^ mol/L) effectively attenuates the severity of this calcification (97–99). This attenuation is likely mediated through the osteoprotegerin, receptor activator of nuclear factor-kappa B ligand, and the receptor activator of NF-κB axis which regulates vascular calcification.

Combined, it is shown that serum ghrelin may be a novel predictor of vascular calcification in diabetic patients and that exogenous administration of ghrelin may be an effective agent in attenuating calcification.

## The Influence of Ghrelin on MicroRNAs (miRs)

MicroRNAs are endogenous, small, non-coding RNAs of ~20–22 nucleotides which regulate gene expression at the post-transcriptional level, by either translational inhibition or by messenger ribonucleic acid cleavage (100, 101). So far, 2,588 mature human miRs have been registered at miRBase in release 21,^1^ which are believed to regulate the expression of 30~50% of human genes (102). Since their discovery in 1993 (103, 104), miRs have gained considerable interest as key modulators in a range of pathological and physiological events (105, 106). A single miR can regulate a plethora of targets and, in doing so, evoke a complex multifactorial physiological process (106). miRs are incredibly stable in circulation due to their transport in small membrane vesicles (exosomes and microvesicles) (107, 108) and, therefore, show promise as clinical biomarkers of disease (105, 109–112). Numerous miRs have been shown to be differentially expressed between PAD/CLI patients and control subjects (113–116). However, the functional significance of these miRs is yet to be truly elucidated. In addition to being biomarkers of disease, miRs have also been shown to be modulated by therapeutic agents.

In murine models of CLI, ghrelin administration has been shown to regulate a variety of miRs (Table 2), leading to a multitude of physiological responses (22, 69). Figure one summarizes the beneficial effects ghrelin has on the expression of several miRs, with the preceding section describing each miR in detail.

**Table 2:** Overview of the confirmed targets for micoRNAs regulated by ghrelin.

MicroRNA	Confirmed targets	Biological processes affected
hsa-miR-126	VCAM1 (117), SPRED1, and PIK3R2 (118–120)	Pro-angiogenic and vascular integrity

hsa-miR-132	p120RasGAP (121)	Pro-angiogenic

hsa-miR-206	VegfAa (122, 123)	Antiangiogenic

hsa-miR-92a	Integrin α5 (124) and SIRT1 (125, 126)	Antiangiogenic

hsa-miR-221/222	p27 (Cdkn1b) (126), p57(CDKN1C) (127), and c-Kit (70)	Muscle regeneration and antiangiogenic

hsa-miR-30a	Snail 1 (128–130)	Anti-fibrotic

hsa-miR-21	Spry1 (131) and PTEN (132)	Pro-fibrotic

*VCAM, vascular cell adhesion molecule; SPRED1, sprouty-related protein 1; PIK3R2, phosphoinositol-3-kinase regulatory subunit 2; VegfAa, Vascular endothelial growth factor Aa; SIRT1, surtuin 1; Spry1, Sprouty homolog; PTEN, phosphatase and tensin homolog*.

### MicroRNA-126

AG has been shown to induce post-ischemic angiogenesis through the up-regulation of miR-126 (22), the most characterized and abundant pro-angiogenic miR in endothelial cells [reviewed in Ref. (117)]. miR-126 resides within intron seven of the epidermal growth factor-like domain, multiple 7 gene on chromosome 9q34.3, and gives rise to two mature miRs; miR-126-3p and miR-126-5p. miR-126-3p has been well studied for its role in vascular remodeling [reviewed in Ref. (133)]. miR-126 is essential for blood vessel growth in zebrafish (118) and mice (119). Specific deletion of miR-126 is 50% embryonic lethal, with surviving mutants expressing fragile, leaky vessels, and severe haemorrhage (120, 134). These findings highlight the fundamental role of miR-126 in regulation of vascular integrity and angiogenesis. Consequently, this fundamental role of miR-126 in the vasculature has led to research investigating its regenerative potential, which was confirmed in several animal models of ischemic vascular injury (135, 136, 137).

Mechanistically, miR-126 targets sprouty-related protein 1 (SPRED1) and phosphoinositol-3-kinase regulatory subunit 2 (PIK3R2/p85β), both of which suppress VEGF, *via* PI3K and Akt signalling pathways, respectively. Thus, miR-126 promotes VEGF expression by targeting several pathways, which promotes an angiogenic response (118).

Des-acylated ghrelin has been shown to exhibit an atheroprotective effect by restoring miR-126 expression. In a murine model of CLI, Des-AG administration increased miR-126, which increased SIRT1 expression and prevented cell senescence via reduced p53 and H3K56 acetylation, protecting deoxyribonucleic acids from damage (71, 138). Furthermore, Des-AG induced an anti-adhesive and anti-inflammatory endothelial phenotype, *via* miR-126 regulated VCAM-1 post-transcriptional regulation (71). Finally, Des-AG administration also restored SOD-2 expression by diminishing ROS production (71).

### MicroRNA-132

#### MicroRNA-132 Is a Highly Conserved miR, Encoded in an Intergenic Region on Chromosome

17p13.3 by the transcription factor cAMP response element-binding protein (CREB) (121). Angiogenic factors such as VEGF and basic fibroblast growth factor lead to phosphorylation of CREB and rapid transcription of miR-132. This transcription suppresses the endothelial GTPase-activating protein p120RasGAP, resulting in Ras activation, and subsequent endothelial cell proliferation and angiogenesis (139). In a murine model of CLI, miR-132/221cluster knockout has been shown to delay perfusion recovery, attributable to the modulation of the Ras-MAPK signalling pathway, a key pathway in neovascularization following ischemia. Katare et al. (22), demonstrated exogenous AG treatment significantly up-regulated miR-132, resulting in angiogenesis in a murine model of CLI, while *in vitro* inhibition of miR-132 reduced the angiogenic potential of AG, evident from decreased proliferation, tube formation, and survival of HUVECs.

### MicroRNA-206

MicroRNA-206 is an evolutionally conserved miR, sharing common expression in muscle from *Caenorhabditis elegans* to human (122). miR-206 is also similar to miR-1, only differing by four nucleotides as mature miRs. miR-206 is found in the intergenic region on chromosome 6p12.2 and has been proposed to be a negative regulator of angiogenesis. This is shown to be through the modulation of the potent angiogenic factor VEGF (122, 123). Further, miR-206 has been shown to specifically suppress VEGF expression in several types of cancer and smooth muscle cells (140), clearly demonstrating the role of miR-206 as a negative regulator of angiogenesis. AG has been shown to suppress miR-206 expression, leading to increased VEGF expression and angiogenesis (22). This indicates that AG up-regulates several pro-angiogenic miRs and down-regulates antiangiogenic miRs leading to a pro-angiogenic environment.

### MicroRNA-92a

MicroRNA-92a is a conserved endothelial cell-specific miR, in the miR-17~92 cluster located at chromosome *13q31.3* (124). miR-92a inhibits endothelial cell sprouting and neovascularization following ischemia, through targeting of the protective endothelial genes integrin α5 and SIRT1 (124, 141). Inhibition of miR-92a has been shown to improve angiogenesis and recovery in murine models of chronic ligation of the anterior descending coronary artery (124), CLI (124), vascular injury (142), and in a porcine model of ischemia/reperfusion injury (143).

Exogenous AG treatment has also shown to decrease miR-92a, which negatively modulates integrin α5, which is crucial for the activation of Akt (144). This leads to improved cell survival through the anti-apoptotic factor B cell lymphoma 2 (145) and improved angiogenesis. Combined, ghrelin’s ability to reduce miR-92a expression leads to further regulation of integrin α5 and SIRT1, both of which critically influence endothelial cell proliferation and migration (146).

### MicroRNA-221/-222 Cluster

The miR-221/-222 cluster is encoded by a highly conserved gene cluster on the human Xp11.3 chromosome (147). miR-221/-222 have been shown to control the differentiation and maturation of skeletal muscle cells, by modulating the protein levels of the cell-cycle inhibitors p27 and p57 (126). Des-AG has been shown to induce skeletal muscle regeneration via SOD-2 expression and miR-221/-222 driven post-transcriptional regulation of the Cip/Kip family members P27^KIP1^ (also known as CDKN1B) and P57^KIP2^ (also known as CDKN1C) (127), leading to satellite cell differentiation and myofiber regeneration (69). This indicates Des-AG may benefit CLI in that it can up-regulate miR-221/-222, leading to skeletal muscle regeneration. However, miR-221/-222 inhibits endothelial cell proliferation and motility, thus blocking angiogenesis by targeting the pro-angiogenic c-kit (70, 147), suggesting that Des-AG may improve the skeletal muscle recovery following CLI, but at the same time may have detrimental effects on the neovasculature.

### MicroRNA-30a

MicroRNA-30a is a member of the miR-30 family, located on chromosome 6q13 (148). miR-30a has recently been shown to play a fundamental role in myocardial (129), peritoneal (130), and hepatic fibrosis (149) through the negative regulation of its target protein Snail 1. AG has been shown to increase miR-30a in a model of CLI, leading to diminished skeletal muscle fibrosis and improved recovery (22).

### MicroRNA-21

MicroRNA-21 is a well characterized pro-fibrotic miR [reviewed in Ref. (150)], located on chromosome 17q23.2, within an intron of the transmembrane protein 49 gene (151). miR-21 promotes fibrosis in several tissues including the heart (132, 151), kidneys (150), and skeletal muscle (152) through fibroblast proliferation by activation of ERK–MAPK pathway (131). Sprouty homolog 1 (SPRY1), a potent inhibitor of the Ras/MEK/ERK pathway, has been shown to be a direct target of miR-21 (131, 153). Thus, miR-21 mediates ERK–MAP kinase activity by means of an effect on SPRY1, leading to fibrosis. Moreover, phosphatase and tensin homolog (PTEN) a well-established target of miR-21, is involved in fibrosis by stimulation of the epithelial-to-mesenchymal transition (132) and interrupts the down- stream activation of Akt, a regulator in fibrotic diseases. Pro-fibrotic TGF-β signalling, which contributes to the progression of fibrosis by promoting fibroblasts activation, has been shown to be regulated by miR-21 *via* a PTEN/Akt-dependent pathway (154). AG has been demonstrated to reduce miR-21 in a model of CLI, thus reducing the TGF-β induced fibrotic signalling cascade (22), leading to decreased fibrosis and improved recovery.

AG and Des-AG exhibit pleiotropic beneficial effects, which all may contribute to improved recovery following CLI. The influence of ghrelin on miRs sheds light on the molecular mechanisms that underpin ghrelin’s therapeutic actions.

## Ghrelin as a Novel Therapeutic Agent

Mounting evidence advocates ghrelin as a novel therapeutic candidate for the treatment of CLI, given ghrelin’ potent ability to repair, restore, and regenerate both the vasculature and skeletal muscle. Despite the vast majority of data presented in this review being from *in vitro* and pre-clinical models, ghrelin has been routinely administered to humans for over a decade with no adverse effects (155). To date, over 400 clinical trials^2^ are registered under the term “ghrelin” in a broad range of conditions. Whether ghrelin can produce the beneficial effects for CLI in humans as presented in this review remains uncertain but is a promising area of clinical research.

To ease to the transition from mouse to man, further investigation of ghrelin biology in humans with PAD will give valuable insight into the ghrelin systems role in the disease. Future pre-clinical studies will also aim to examine ghrelin administration in an overt diabetic model of CLI, adding to the evidence base for the use of ghrelin mimetics for treating CLI and similar conditions. Ghrelin has also recently been implicated as a therapy for numerous other diseases [see Ref. (59) for a review]. However, few human studies have truly explored its therapeutic potential, leaving an apparent gap in the literature into its efficacy as a treatment option for human disease.

## Concluding Remarks

**Figure 2:**
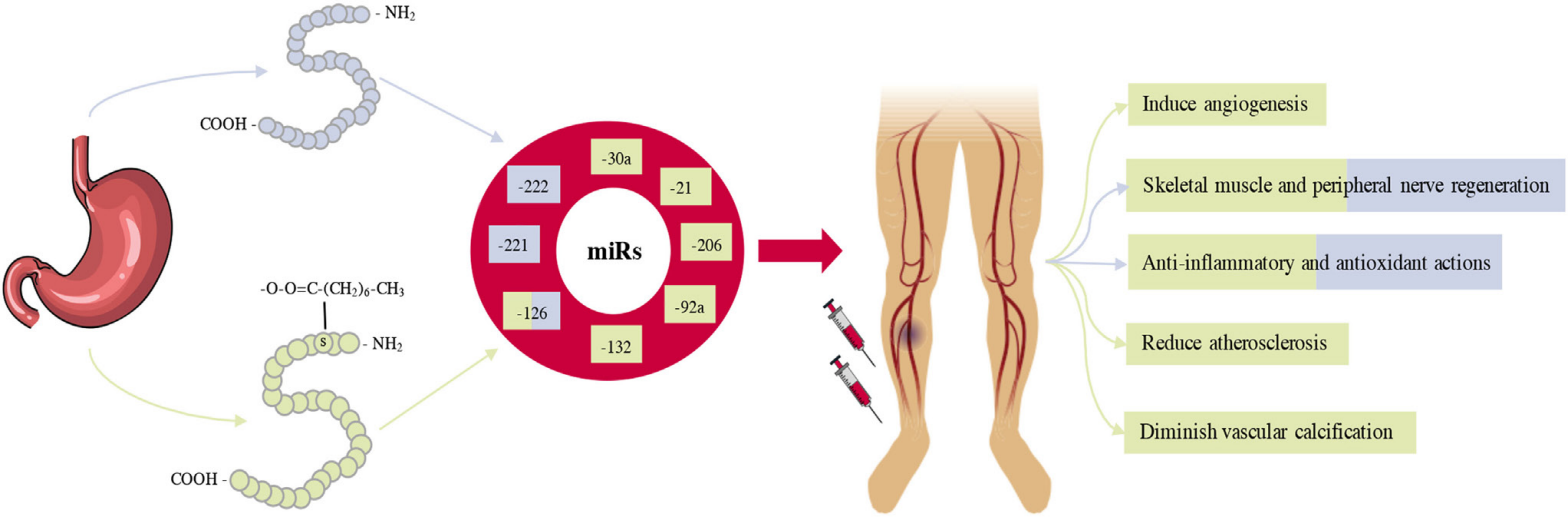
A schematic overview summarizing the role of ghrelin administration in the treatment and management of critical limb ischemia (CLI). Ghrelin is predominantly produced in the stomach and circulates in two forms, Acylated ghrelin (AG) and des-acylated ghrelin (Des-AG). Most of the biological effects of AG and Des-AG in pre-clinical models of CLI appear to be closely related to alterations in several pro-survival microRNAs (miRs). Given ghrelin administration can regulate several miRs, which in turn control a significant proportion of genes, ghrelin administration can result in improved limb perfusion, muscle quality, and ultimately, survival.

In this review, we have described the multifactorial roles of ghrelin in CLI, as demonstrated by its role in multiple regenerative and regulatory processes. Post-ischemic vascular and skeletal muscle remodeling is stimulated by ghrelin, while it inhibits antiangiogenic and pro-fibrotic signalling pathways. With the incidence of CLI continuing to increase, driven by the rapid increase in DM and population aging, the resulting global socio-economic burden of CLI is significant. Current treatment options for CLI patients are costly and often met with poor success, while emerging novel angiogenic agents have limited success in large clinical trials. If the global burden of CLI is to be tackled, novel therapeutic strategies are urgently required. Collectively, the identification and characterization of novel biological properties of ghrelin opens the door for its administration to be a potential therapeutic agent in the treatment of CLI (Summarised in Figure 2).

The pleiotropic actions of ghrelin as presented in this review will hopefully stimulate further clinical studies to explore the potential of ghrelin as a novel therapeutic agent for the treatment of this highly morbid disease.

## Author Contributions

JN performed extensive literature search and wrote the first draft of the review; JP critically reviewed the manuscript; DS and RK equally contributed to conception and design of the manuscript and critically reviewed the drafts of the manuscript. All authors read and approved the final manuscript.

## Conflict of Interest Statement

The authors declare that the research was conducted in the absence of any commercial or financial relationships that could be construed as a potential conflict of interest. The reviewer HK declared a shared affiliation, with no collaboration, with one of the authors, JP, to the handling Editor.

## Footnotes

^1^http://www.mirbase.org.

^2^https://clinicaltrials.gov.
